# Solid-State Phase Transformations in Thermally Treated Ti–6Al–4V Alloy Fabricated via Laser Powder Bed Fusion

**DOI:** 10.3390/ma12182876

**Published:** 2019-09-06

**Authors:** Paolo Mengucci, Eleonora Santecchia, Andrea Gatto, Elena Bassoli, Antonella Sola, Corrado Sciancalepore, Bogdan Rutkowski, Gianni Barucca

**Affiliations:** 1 Dipartimento di Scienze e Ingegneria Della Materia, Dell’Ambiente de Urbanistica, Università Politecnica delle Marche, 60131 Ancona, Italy; 2 INSTM–Consorzio Interuniversitario Nazionale per la Scienza e Tecnologia dei Materiali, 50121 Firenze, Italy; 3 Dipartimento di Ingegneria “Enzo Ferrari”, Università di Modena e Reggio Emilia, 41125 Modena, Italy; 4 Dipartimento di Ingegneria e Architettura, Università di Parma, 43121 Parma, Italy; 5 Faculty of Metals Engineering and Industrial Computer Science, AGH University of Science and Technology, 30-059 Krakow, Poland

**Keywords:** Ti alloys, phase transformations, additive manufacturing, thermal treatments, neutron diffraction, X-ray diffraction, scanning transmission electron microscopy, energy-dispersive spectroscopy

## Abstract

Laser Powder Bed Fusion (LPBF) technology was used to produce samples based on the Ti–6Al–4V alloy for biomedical applications. Solid-state phase transformations induced by thermal treatments were studied by neutron diffraction (ND), X-ray diffraction (XRD), scanning transmission electron microscopy (STEM) and energy-dispersive spectroscopy (EDS). Although, ND analysis is rather uncommon in such studies, this technique allowed evidencing the presence of retained β in α’ martensite of the as-produced (#AP) sample. The retained β was not detectable by XRD analysis, nor by STEM observations. Martensite contains a high number of defects, mainly dislocations, that anneal during the thermal treatment. Element diffusion and partitioning are the main mechanisms in the α ↔ β transformation that causes lattice expansion during heating and determines the final shape and size of phases. The retained β phase plays a key role in the α’ → β transformation kinetics.

## 1. Introduction

Metal additive manufacturing is now taking the lead in market sections where low production volumes, freedom of design, and a high level of customization are the guiding lights, such as in biomedical and aerospace markets [1,2]. Furthermore, this manufacturing technique is particularly suitable for strategic metallic materials, which are difficult and expensive to manufacture with traditional subtractive technologies. Titanium and its alloys are the most representative example, being advanced structural materials, characterized by outstanding properties such as high strength-to-weight ratios, fracture toughness, corrosion resistance (immunity to seawater), and biocompatibility, but also affected by major technological drawbacks in terms of machinability, such as the extreme reactivity and the low thermal conductivity [3,4]. Only low-speed machining of forged titanium has been successfully employed over history, resulting in more than half of the raw materials wasted as scrap [5,6].

The α + β titanium alloy Ti–6Al–4V is the most studied in terms of metallic materials processable by additive manufacturing (AM) and, in particular, by the Powder Bed Fusion (PBF) technique [7]. Starting from a computer-aided design (CAD) file, a high-energy beam, typically a laser or an electron beam, selectively fuses metal powder particles layer-upon-layer, in order to build a three-dimensional (3D) object, while the surrounding nonirradiated powder acts as support for the built part as well as for the following layers.

The fast cooling rates (10^4^–10^6^ K/s) of the small melt pools typical of Laser Powder Bed Fusion (LPBF) exceed the critical cooling rate required for martensitic transformations in Ti–6Al–4V [8], resulting in phase transformation of the high-temperature bcc β-Ti phase into the metastable hcp α’-Ti phase through a shear-type diffusionless process. This gives rise to an as-built microstructure of elongated prior β grains filled with fine α’ martensite [9,10]. Properties of materials are linked to microstructural features including phases and their length scale, morphology, and distribution, therefore the poor ductility typical of as-built LPBF samples is induced by the combined detrimental effect of intergranular failure due to the nonisometric shape of prior β grains and the hindered dislocation motion attributed to the tightly spaced fine martensitic grains [11,12]. However, the same microstructure shows strength values which are comparable or, in some cases, better than the same alloy produced by traditional manufacturing techniques, and the application of heat treatments allows to induce a phase transition from the martensitic α’ to a coarse α + β, which improves the ductility but lowers strength, leading to the so-called strength–ductility dilemma [13,14]. 

In recent years, a number of research efforts concentrated on the optimization of the LPBF process parameters such as input energy, hatch distance, layer thickness, scan speed, scan strategy, and focal offset distance [15,16,17,18], and on further post-processing heat treatments to obtain peculiar microstructures and mechanical properties, according to the targeted application.

In the field of biomaterials, and particularly in orthopedics and dentistry applications, the LPBF technology is mostly appreciated for its high degree of individualization and the capability of producing low-volume devices and complex geometric features in a single production process. However, during the LPBF process of metal alloys, new and unexpected nanostructures [19] as well as novel precipitates stoichiometry and precipitation sequence [20] can develop, that strongly modify the mechanical performances of parts. Therefore, accurate characterization of LPBF-produced devices is necessary to clarify the structural mechanisms at the nanoscale responsible for the macroscopic behavior.

In this study, a Ti–6Al–4V alloy has been produced by LPBF, following the material processing used in the production of commercial devices employed in dentistry. In particular, after the LPBF process, samples were submitted to a five-step heat treatment to simulate the veneering of metals parts with ceramics, commonly used in dentistry for aesthetic reasons. Results concerning structural characterization of raw powder, as-produced sample, and thermally treated sample, as well as results of the mechanical tests performed on the same samples have been reported in a previous paper [21].

The aim of the present paper is to investigate phase transitions induced by thermal treatments performed on Ti–6Al–4V samples produced by LPBF. Structural characterization has been carried out by X-ray diffraction (XRD), scanning transmission electron microscopy (STEM), energy-dispersive spectroscopy (EDS), and neutron diffraction (ND). Among the different analytical techniques used in this study, ND proved to be the only one capable of detecting the presence of retained β-Ti in α’-Ti martensite after the LPBF production process. The presence of a small amount of retained β-Ti in α’-Ti martensite is generally difficult to detect by conventional techniques such as XRD and TEM. In this paper, for the first time, an approach based on neutron diffraction (ND) is proposed in order to overcome difficulties related to detection of retained β-Ti in α’-Ti martensite. Results show that the retained β phase is responsible for the peculiar microstructure observed after the five-step heat treatment. 

## 2. Materials and Methods

### 2.1. Material and Production Process

Laser Powder Bed Fusion (LPBF) technology was used to produce test samples in an EOSINT M290 system equipped with a solid-state Yb fiber laser operating in the following conditions: (a) Laser beam power 280 W, (b) Laser spot diameter 200 μm, (c) Laser scan speed 1200 mm/s, (d) Hatch distance 0.14 mm, (e) layer thickness 30 μm, and (f) chess scan strategy. The LPBF process was carried out in argon atmosphere.

The raw material was virgin new and originally sealed EOS Ti64 powder, supplied by EOS GmbH Electro Optical System, with chemical composition compatible with the Ti–6Al–4V alloy, corresponding to ISO 5832-3, ASTM F1472, and ASTM B348. The nominal composition of the EOS Ti64 powder, as reported in the material datasheet from the manufacturer, is shown in Table 1. Further details on LPBF parameters, powder properties, and composition of parts before and after thermal treatments can be found elsewhere [21]. From now on, samples of the raw powder are indicated as #PW.

Parallelepiped samples with size 31.8 mm × 6.4 mm × 12.7 mm (X × Y × Z) were produced (X is the slide direction of the recoater, and Z is the growth direction). Immediately after production, samples were submitted to thermal treatment at 800 °C for 4 h in argon atmosphere to reduce material anisotropy due to the layer-by-layer building method. As this annealing thermal treatment is part of the production process and considering that devices produced from the EOS Ti64 powder are commercialized in this state, from now on, samples after the above-described treatment are referred to as “As Produced” (#AP).

### 2.2. Thermal Treatments

In order to investigate the α ↔ β transformation behavior, samples in the #AP condition were submitted to a five-step thermal treatment, compliant to EN ISO 22674, which simulates the firing cycle commonly used in dental applications to veneer metallic devices based on the Ti–6Al–4V alloy with dental ceramic materials. The thermal treatment, starting from a preheating temperature of 420 °C, consists of a series of five heating and cooling ramps performed in vacuum that heat the sample up to 945 °C [21]. Samples submitted to this thermal treatment, from now on, are indicated as “#TT”.

### 2.3. Mechanical Tests

Mechanical tests consisting of flexural tests as well as hardness and roughness measurements were performed on both #AP and #TT samples, results have been discussed elsewhere [21].

### 2.4. Characterization Techniques

Structure characterization was performed by neutron diffraction (ND), X-ray diffraction (XRD), scanning transmission electron microscopy (STEM) in both bright-field (BF) and dark-field (DF) mode, and energy-dispersive X-ray microanalysis (EDS).

Neutron diffraction investigation was performed in the scattering vector range Q = 1.5 − 7.5 Å^−1^ by using facilities available at the Budapest Neutron Centre (BNC).

XRD measurements were carried out by a Bruker D8 Advance X-ray diffractometer in the angular range 2θ = 10° − 85°, corresponding to the vector scattering range Q = 0.7 − 5.5 Å^−1^, by using Cu–Kα radiation (λ = 0.154056 nm). Analysis of the XRD patterns performed by Rietveld refinement using the MAUD (Materials Analysis Using Diffraction) software allowed estimating of lattice parameters and relative content of the Ti phases present in the samples [22]. Shape analysis of peaks as well as peak deconvolution were obtained by the Origin software package [23]. All peaks present in the XRD patterns were indexed with reference to the International Centre for Diffraction Data (ICDD) files for low-temperature hexagonal (hcp) α-Ti (44-1294) and high-temperature cubic (bcc) β-Ti (44-1288) phases.

A probe C_s_-corrected FEI Titan^3^ G2 60-300 equipped with ChemiSTEM technology was used for STEM and HRSTEM observations and EDS analysis. Lamellae for STEM and EDS analyses were prepared by a ZEISS NEON CrossBeam 40EsB Focused Ion Beam (FIB) system. Before milling, a layer of Pt was deposited at the place of cutting in order to protect the thin sample against heavy Ga ions during preparation. Final milling was performed by 4 keV Ga + ion beam.

## 3. Results

Results obtained from the different analytical techniques used in the present study are reported separately.

### 3.1. Neutron Diffraction (ND)

**Figure 1:**
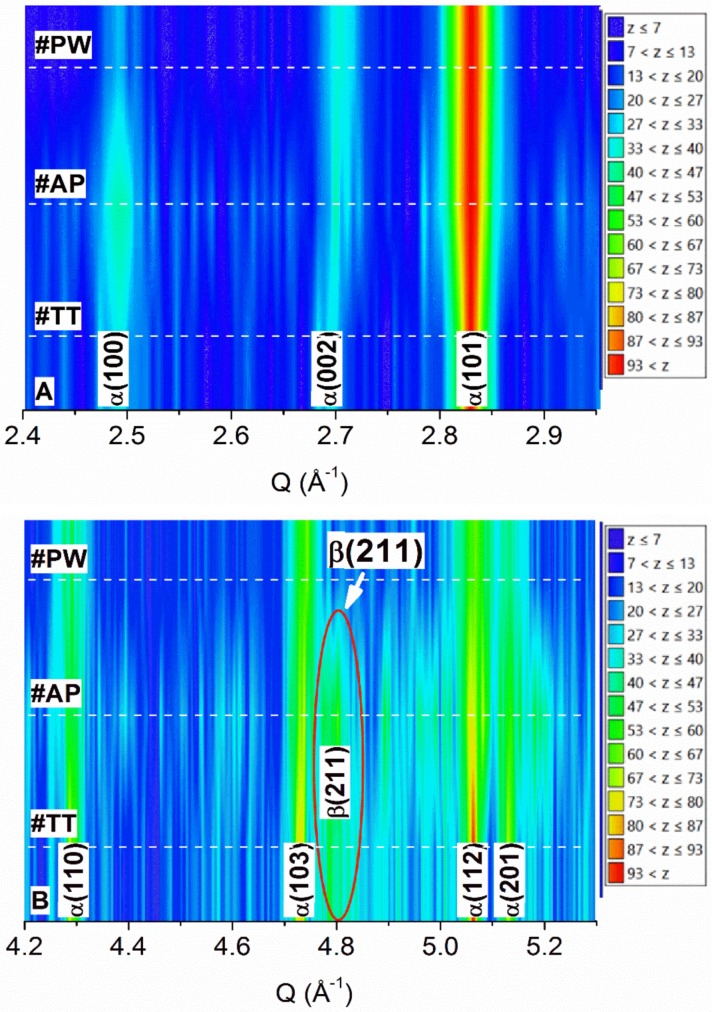
Neutron diffraction (ND) intensity color map of the three investigated samples for two different ranges of the scattering vector Q: (**A**) low-value range Q = 2.4 − 2.95 Å^−1^; (**B**) high-value range Q = 4.2 − 5.3 Å^−1^. Miller indices of peaks from α-Ti and β-Ti phases are reported. The color code on the right of figures indicates peak intensity range. Diffraction effect due to the β-Ti high-temperature phase in samples #AP and #TT is evidenced by the red ellipse in Figure 1B.

The intensity color map of neutron diffraction for the three analyzed samples is reported in Figure 1. The color code on the right of Figure 1A,B indicates peak intensity range. Two different ranges of the scattering vector Q were investigated: low-value range Q = 2.4 − 2.95 Å^−1^ where the most intense diffraction peaks of Ti are located (Figure 1A), and high-value range Q = 4.2 − 5.3 Å^−1^ containing low-intensity peaks (Figure 1B). In the color maps, peak position is indicated by the Miller indices of the corresponding Ti phase, while dashed lines mark the exact position of patterns from #PW, #AP and #TT samples.

In Figure 1B, the red ellipse indicated by arrow evidences diffraction effects due to (211) lattice planes of the cubic (bcc) high-temperature β-Ti phase in samples #AP and #TT. This latter effect is completely absent in sample #PW (Figure 1B).

Diffraction peaks in sample #AP (Figure 1A) are generally broad and weak, becoming more defined and sharper after the thermal treatment (#TT). This effect is pronounced for the α(100) peak of sample #AP (Figure 1A).

**Figure 2:**
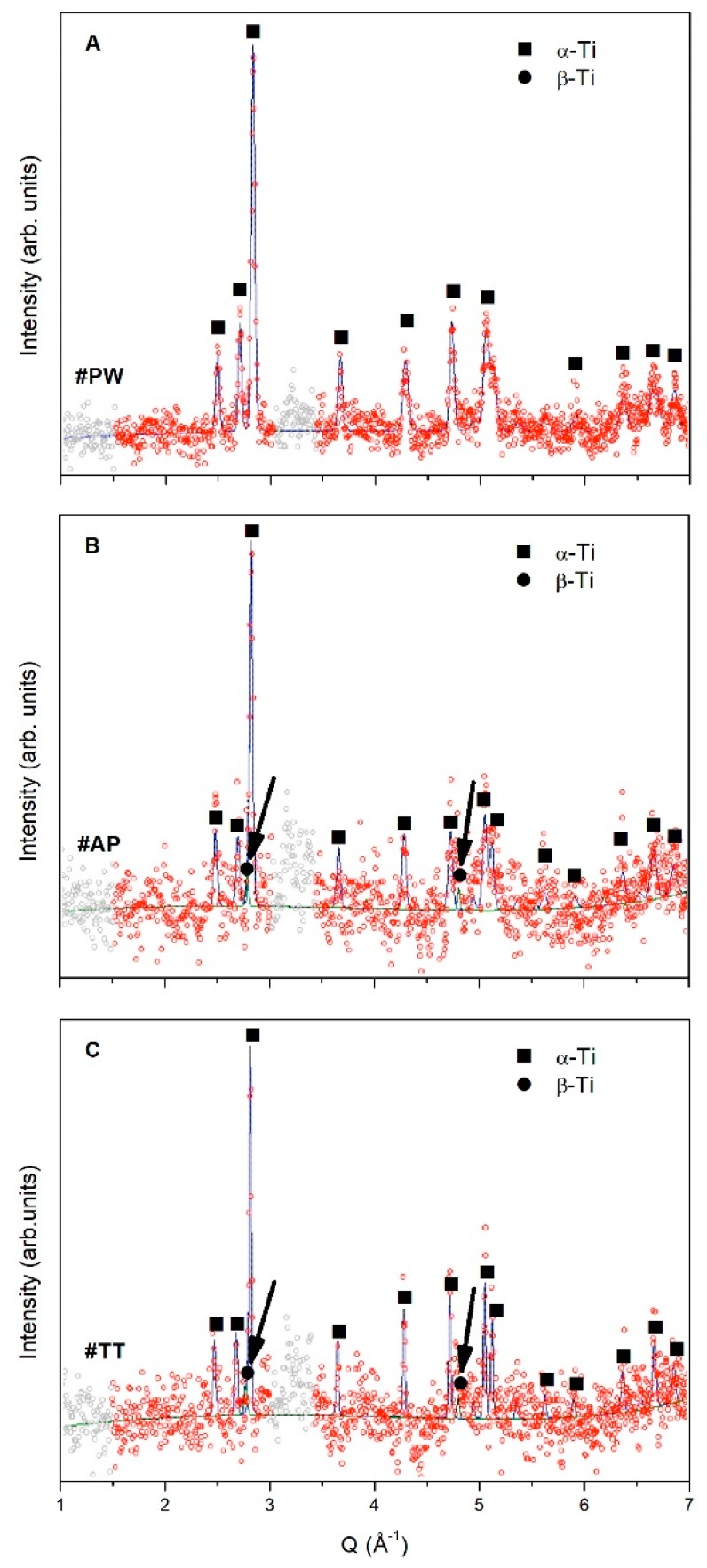
ND patterns of samples: (**A**) powder (#PW), (**B**) as-produced (#AP) and (**C**) thermal-treated (#TT). Experimental data points: red dots; Rietveld refining: continuous line. Gray regions of patterns are excluded Q ranges due to instrument background. Full square: α-Ti; Full dots: β-Ti. Arrows indicate peak position of the β-Ti phase.

In order to further achieve quantitative information from ND patterns, Rietveld refinement was carried out, results are reported in Figure 2. Experimental data points (red dots) in Figure 2 are fitted by Rietveld analysis program (continuous line), while grey dots in the pattern indicate excluded regions due to instrument background. Diffraction peaks from the α-Ti phase are indicated by full squares, while full dots and arrows indicate diffraction peaks due to β-Ti (Figure 2).

Powder (#PW) is entirely formed of α-Ti (Figure 2A). On the contrary, the LPBF sample in the as-produced condition (#AP) as well as the thermally treated sample (#TT), in addition to the α-Ti phase contain also the high-temperature β-Ti phase (Figure 2B,C). Estimation of lattice parameter of the β-Ti phase from ND patterns provided a_β_ = 0.31973 nm for the #AP sample and a_β_ = 0.32037 nm for #TT. Lattice parameters of the α-Ti phase are compatible with the XRD results reported below.

### 3.2. X-ray Diffraction (XRD)

**Figure 3:**
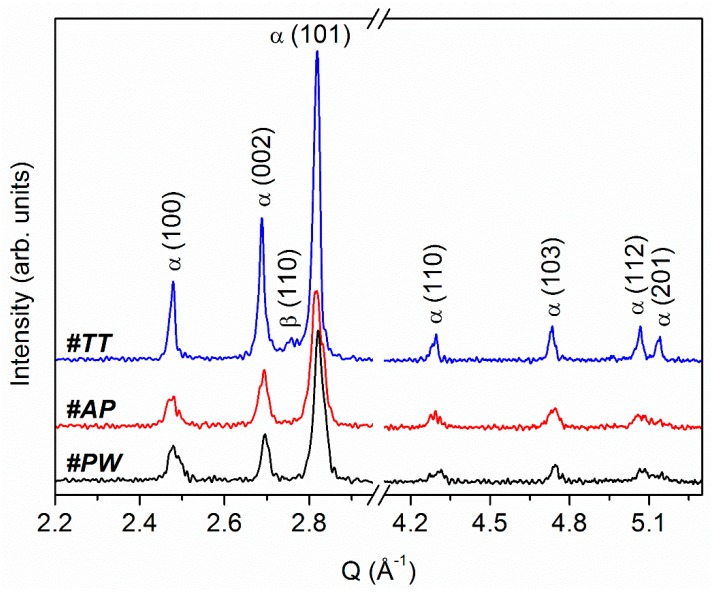
XRD patterns of samples in the same scattering vector range of neutron diffraction analysis.

XRD results obtained in the same low and high Q value ranges as ND are shown in Figure 3. In this case, results are reported in function of Q, the scattering vector, to ease comparison between XRD and ND experimental patterns.

XRD clearly shows presence of α-Ti in the pattern of all samples, while β-Ti is evidenced as a weak and broad peak around Q = 2.75 Å^−1^ (2θ = 39.459°) exclusively in the pattern of heat-treated (#TT) sample (Figure 3).

Rietveld refining of XRD patterns allowed calculation of the lattice parameters of Ti phases present in the analyzed samples.

All results obtained from ND and XRD analysis are summarized in Table 2, where reference values from the ICDD files are reported for comparison. 

Values of lattice parameters for both α-Ti and β-Ti phases are always lower than reference, independently of sample type (Table 2). A slight increase in lattice parameters of both phases is observed as a consequence of thermal treatment. The lattice parameters values obtained from XRD and ND are in close agreement.

Volume fraction of β-Ti in the #TT sample, as estimated by Rietveld refining of XRD pattern, is about 7%.

### 3.3. Scanning Transmission Electron Microscopy (STEM) and Microanalysis (EDS)

**Figure 4:**
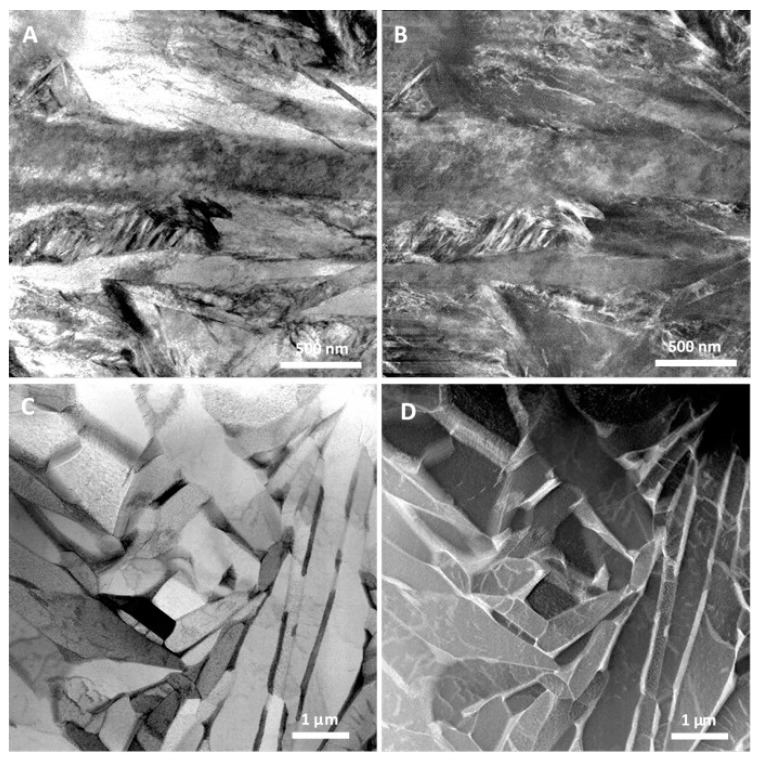
Scanning Transmission Electron Microscopy (STEM) images of samples #AP and #TT in bright-field (BF) and dark-field (DF) mode: (**A**) #AP–BF; (**B**) #AP–DF; (**C**) #TT–BF; (**D**) #TT–DF.

STEM investigations of samples #AP and #TT are shown in Figure 4. High density of crystallographic defects, mainly dislocations, is visible in the STEM images of sample #AP both in bright-field (BF) mode (Figure 4A) and in dark-field (DF) mode (Figure 4B) of the same sample zone. 

Elongated grains resembling a martensitic structure typical of high cooling rates constitutes the material microstructure after the LPBF process.

After thermal treatment, grains appear more regular in shape, although regions with elongated grains resembling the previous martensitic structure are still visible (Figure 4C). However, dislocation density inside grains is now sensibly lower with respect to the #AP sample, with a tendency to reorganize in low-angle subgrain boundaries (Figure 4C). The dark-field (DF) image of the same area of the #TT sample is shown in Figure 4D. The DF image, taken by the high-angle annular detector of the STEM equipment, evidences compositional contrast. Therefore, the elongated brighter zones preferentially located at the grain boundaries in Figure 4D contain higher atomic number elements with respect to the adjacent darker regions. Electron-diffraction investigations performed on different areas of the #TT sample showed that grains are mainly formed of α-Ti, while the elongated zones at the grain boundaries are β-Ti [21]. It is worth noting that the β-Ti phase is discontinuous around the α-Ti grain, giving rise to a peculiar microstructure.

**Figure 5:**
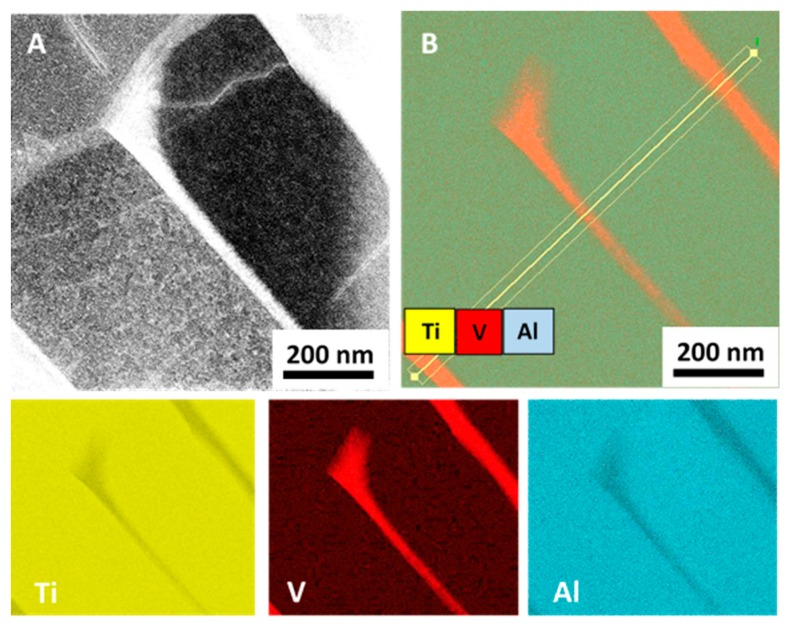
Energy-dispersive spectroscopy (EDS) analysis of sample #TT in correspondence to three grain boundaries: (**A**) STEM Dark-Field image; (**B**) EDS map for Ti, Al, and V. EDS maps of single elements are also reported for Ti, V, and Al.

EDS analysis mapping of the #TT sample allowed evidencing of element distribution inside the different regions. Results obtained in correspondence to a grain boundary are reported in Figure 5. 

Figure 5A is the DF image of the investigated zone, and Figure 5B is the corresponding EDS map performed considering Ti, Al, and V. EDS maps of single elements (Ti, V, Al) are also reported in Figure 5.

**Figure 6:**
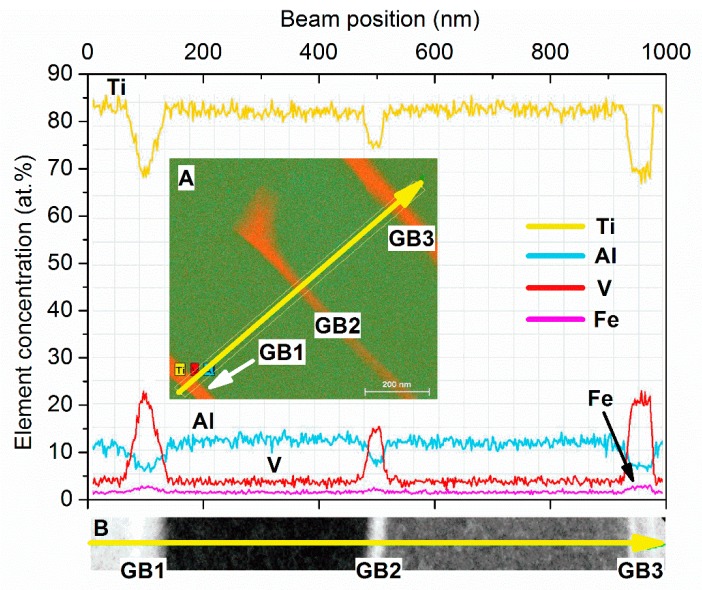
Element concentration (in at.%) obtained by EDS line-scan analysis. Inset A: location of the scanned line (arrow) on the sample. Inset B: scanned line (arrow) with the position of grain boundary (GB) marked.

In order to quantify element concentration inside the different regions evidenced in Figure 5, EDS in line-scan analysis was carried out in the same area, results are reported in Figure 6.

Figure 6 reports concentration (in at.%) of Ti, V, Al, and Fe along the scanned line. The inset A in Figure 6 shows the exact location of the scanned line (arrow) on the sample. The direction of the line scanning is indicated by arrow (from tail to tip). The scanned line intercepts three grain boundaries (GB) marked as GB1, GB2, and GB3, respectively. Element concentration profile along the scanned line for each element is given in at.%. The graph scale in Figure 6 measures the distance (in nm) along the scanned line from the starting point (arrow tail). The uppermost curve (Ti) in Figure 6 shows variation of Ti concentration along the scanned line. Curves in the lower part of Figure 6 report concentration of Al, V, and Fe, respectively. The inset B, located below the bottom axis of the graph, is the STEM dark-field image of the region analyzed. Location and direction of the scanned line is indicated by arrow, while the positions of the three grain boundaries GB1, GB2, and GB3 shown in inset A are marked for reference. 

Results clearly show a strong increase of V concentration at grain boundaries (GB1, GB2, and GB3) in correspondence to β-Ti. In particular, V concentration in β-Ti reaches 23 at.% (about 25 wt.%), while Al and Ti decrease to 7 at.% (about 4 wt.%) and 70 at.% (about 71 wt.%), respectively. Furthermore, the increase of V in β-Ti is always accompanied by a slight increase of Fe (Figure 6).

## 4. Discussion

The EOS Ti64 powder consists of spherical particles with diameter size ranging from 0.7 μm to 118 μm and average diameter of 16 μm [21]. Powder was processed in the EOSINT M290 system by using the LPBF parameters reported above. Structural characterization of the raw powder performed by ND (Figure 1 and Figure 2A) and XRD (Figure 3) shows single phase α-Ti with lattice parameter values slightly lower than those of the reference (Table 2). Reduction amounts to about 0.7% and 0.4% for the lattice parameters a and c, respectively. Accordingly, the volume of the α-Ti hexagonal unit cell results about 1.8% lower than that of the reference. The difference in lattice parameters of α-Ti is due to the atomization process used for powder production, which involves high cooling rates [24]. Therefore, both ND and XRD investigations show a well crystallized α-Ti single phase powder.

After the LPBF process (#AP), diffraction peaks in ND and XRD patterns show a general trend to increase width and reduce height. The ND intensity color map in Figure 1 clearly shows this trend, which is further confirmed by the ND pattern in Figure 2B and the XRD pattern in Figure 3. Lower intensity and larger width of diffraction peaks are associated to high defective structure. STEM images in Figure 4A,B confirm high density of defects in the martensitic structure of sample #AP. Formation of martensite during AM processing of Ti–6Al–4V alloy is well known in literature and was attributed to the high cooling rates developed during the LPBF process [8,9,10]. Comparison of STEM images in BF and DF mode (Figure 4A,B) of the same sample zone evidences high density of defects, mainly dislocations, inside grains of the martensitic structure.

Although, ND and XRD provide results in general agreement in terms of phases and lattice parameters, in the case of sample #AP they show marked discrepancies. In particular, while ND shows a diffraction effect in correspondence of the (211) peak position of the high-temperature cubic β-Ti phase (Figure 1B), XRD pattern does not evidence any effect attributable to this phase (Figure 3). Malinov and Sha [25] have already reported on difficulties of detecting small amounts of β-Ti in Ti–6Al–4V alloy by using conventional XRD and TEM techniques. They have also evidenced that this difficulty is further enhanced in the presence of martensitic α-Ti (commonly referred to as α’-Ti). In order to overcome difficulty of β-Ti detection, Malinov and Sha proposed synchrotron radiation experiments [26]. Recently, Cho et al. [16] performed XRD measurements in microdiffraction mode (μXRD) by using a laboratory equipment, demonstrating the possibility of detecting low-intensity diffraction effects attributable to β-Ti in as-produced parts with predominant α’/α-Ti microstructure. This latter result obtained by μXRD was ascribed to the small volume fraction of β-Ti, that cannot be detected by conventional XRD, nor by TEM, as already suggested by Malinov and Sha [25]. Therefore, most papers in literature reporting single-phase α’/α-Ti composition of produced parts that based conclusion on XRD and/or TEM evidences, may have neglected the presence of primary or retained β-Ti, which play a key role in the α ↔ β transformation during subsequent heat treatments.

In this paper, for the first time, an approach based on neutron diffraction (ND) is proposed in order to overcome difficulties related to detection of retained β-Ti in α’-Ti martensite.

Neutrons interact with matter through nuclear interactions, while X-rays interact through electromagnetic interactions with the electron cloud of atoms. In neutron scattering, the atomic nuclei are point particles, while in X-ray scattering, atom size is comparable to the wavelength of probing radiation. For this reason, neutrons have high penetration (low absorption) for most elements, making neutron scattering a bulk probe. Furthermore, neutrons have the right momentum and energy transfer to investigate both structures and dynamics in condensed matter [27,28].

In this study, we take advantage of the larger volume investigated by ND to increase the diffraction effects from the β-Ti phase, which in this way become detectable. As a result, ND provides clear evidence of retained β-Ti in the #AP sample (Figure 1B and Figure 2B), which otherwise is prevalently constituted of α’-Ti martensite (Figure 4A,B). Lattice parameter of the retained bcc β-Ti, estimated from the ND pattern in Figure 2B, is about 3.4% lower than the reference value (Table 2). This effect could be ascribed to the high cooling rates typical of the AM processes that are responsible for the high defective martensite. Ahmed and Rack [29] have demonstrated that cooling rates above 410 K/s result in a fully martensitic microstructure. On the other hand, in AM processes, typical values of cooling rate exceed 10^4^ K/s [7,8], thus justifying the martensitic α’-Ti structure observed in the #AP sample. However, as evidenced by Kirka et al. [30], on increasing the number of layers deposited in the AM process, α’ can completely decompose into α+β or undergo a partial decomposition to α’ + α + β microstructure. In particular, the top layer sintered by the laser action undergoes a phase transformation that can be summarized as liquidus → β → α’, while in most of the underlying layers, solid-state phase transformations occur that cause multiple cycles of diffusionless β ↔ α’, resulting in a final mixture of α’, α, and β phases [30].

In complete agreement with Kirka et al. [30], our results obtained from ND and XRD investigations on the #AP sample evidence a mixture of highly defective α’-Ti martensite and retained β-Ti phase.

Thermally treating the highly defective microstructure observed in sample #AP induces elements diffusion and defects annealing. Diffusion of elements is strongly favored by defects, which therefore have a role in the solid-state phase transformations, as already shown by Haubrich et al. [31]. Nevertheless, diffusion induces element partitioning that is substantially the basics of martensite decomposition: Al and O are accumulated in the α/α’ phase, while V and Fe accumulate in the β phase [31,32].

During martensite decomposition, lattice variations of both α/α’ and β phases occur together with stress relaxation and defects annealing, resulting in final lattice parameters of phases dependent on cooling rates [33].

In our case, ND and XRD results for the #TT sample evidence a slight increase of both α and β phases lattice parameters (Table 2) with respect to #AP. The lattice expansion of α and β phases on thermal treatments has been deeply investigated by Elmer et al. [33], who found an increase of the average thermal expansion coefficient during the α → β transformation on annealing. However, they also observed an anomaly decreased expansion of the β phase at around 500–600 °C that attributed to an increase of the V concentration. The same effect is reported in the paper by Huabrich et al. [31] in a sample annealed at 400 °C for 2 h.

Considering that our alloy has been treated in the temperature range 500–945 °C, well above the critical temperature for the anomalous behavior of the expansion coefficient, the increase in lattice parameters of the β phase measured in the #TT sample can be attributed to V incorporation inside the cubic structure.

As shown in Figure 5 and Figure 6, the β-Ti phase formed at the grain boundaries of the α-Ti phase is discontinuous and greatly enriched in V.

As a consequence of annealing, dislocation density inside grains is dramatically reduced during the thermal treatment (Figure 4C,D), thus grain boundaries remain the energetically favored paths for elements diffusion. This latter effect also justifies the elongated shape of β phase, which grows following the grain boundary line, with a limited width in direction perpendicular to the grain boundary (Figure 4C,D). The fast diffusion of elements along grain boundaries gives rise to accumulation of V in the β phase, which reaches concentrations as high as 23 at.% (about 25 wt.%), as observed in Figure 6. It is worth noting that the strong increase in V concentration in the β phase is always accompanied by a corresponding increase in Fe content (Figure 6), confirming element partitioning as the basic mechanism in α ↔ β transformations.

Haubrich et al. [31], by atom probe tomography (APT), found two-dimensional features with thickness below ~3 nm that they considered as possible precursors for precipitation of β within α’ laths.

In our case, is the retained β phase, evidenced by ND investigations after production (sample #AP), plays a key role in the α’ → β transformation kinetics during heating, justifying the peculiar microstructure observed after the thermal treatment (sample #TT). In fact, during heating, the retained β phase located at the grain boundary or in its proximity coarsens and stores V by the mechanism described above, without any nucleation process involved, resulting in an accelerated transformation kinetics. Therefore, although formation of β-Ti by the slower nucleation and growth mechanism also occurs during heating, the retained β phase at the grain boundary grows faster at the expense of the surrounding α’, giving rise to the discontinuous peculiar microstructure observed in the #TT sample. Furthermore, due to vanadium incorporation, the lattice parameter of the β-Ti cubic phase increases, as evidenced by ND analysis.

## 5. Conclusions

Phase transformations induced by heat treatments in a Ti–6Al–4V alloy were investigated by neutron diffraction (ND), X-ray diffraction (XRD), scanning transmission electron microscopy (STEM), and energy-dispersive microanalysis (EDS). The alloy, specifically developed for biomedical applications, was produced by laser powder bed fusion (LPBF), following the material processing used in the production of commercial devices employed in dentistry. 

Neutron diffraction (ND) is not commonly used in this kind of study and, to our best knowledge, this is the first time that ND has been employed to investigate the alloy microstructure to follow phase evolution during thermal treatments.

The main results obtained can be summarized as follows:

- The raw powder is composed of single-phase α-Ti;
- The as-produced sample (#AP) is formed of highly defective α’-Ti martensite with retained β-Ti phase (evidenced only by ND);
- The thermally treated sample (#TT) has more regularly shaped grains, with defects density sensibly reduced and elongated β-Ti phase at the grain boundaries;
- The β-Ti phase accumulates high quantity of V (up to 25 wt.%) during annealing due to element diffusion and partitioning;
- Increase of V content is always accompanied by an increase of Fe.

Element diffusion and partitioning are the main mechanisms in the α ↔ β transformation that influence lattice expansion during heating as well as final shape and size of phases.

The retained β-Ti phase plays a key role in the kinetics of the α’ → β transformation during heating, thus justifying the peculiar microstructure observed in the thermally treated sample.

## Figures and Tables

**Table 1:** Nominal composition of EOS Ti64 powder from the material datasheet.

Al (wt.%)	V (wt.%)	O (ppm)	N (ppm)	C (ppm)	H (ppm)	Fe (ppm)	Ti
5.50–6.75	3.50–4.50	<2000	<500	<800	<150	<3000	Balance

**Table 2:** Lattice parameters of Ti phases as estimated from XRD and ND investigations. Values from International Centre for Diffraction Data (ICDD) files (α-Ti 44-1294, β-Ti 44-1288) are shown as reference.

Ti Phase	#PW	#AP	#TT	Reference
α	a = 0.29291 nm (XRD) c = 0.46631 nm (XRD)	a = 0.29222 nm (XRD) c = 0.46604 nm (XRD)	a = 0.29274 nm (XRD) c = 0.46742 nm (XRD)	a = 0.29505 nm c = 0.46826 nm
β	not detected (XRD) not detected (ND)	not detected (XRD) a = 0.31973 nm (ND)	a = 0.32259 nm (XRD) a = 0.32037 nm (ND)	a = 0.33065 nm
